# Mapping malaria risk using geospatial and AHP approaches in Nsanje District, Malawi

**DOI:** 10.1017/S0950268826101678

**Published:** 2026-05-21

**Authors:** Yanjanani Miston Banda, Jabulani Nyengere, Kimberly Saka, Harineck Tholo, Chikondi Chisenga, John Njalammano, Blessings Nthezemu Kamanga, Steven Gondwe, Andrew G. Mtewa, Amon Abraham, Lemson Kachedwa, James Chirombo, Alick Nguvulu, Emmanuel Chinkaka, Wilfred Kadewa, Richard Lizwe Steven Mvula

**Affiliations:** 1 Department of Earth Sciences, Malawi University of Science and Technology, Malawi; 2 Department of Water Resources, Malawi University of Science and Technology, Malawi; 3 Global Health Informatics Institute, Malawi; 4 Department of Applied Studies, Chemistry Section, Malawi University of Science and Technology, Malawi; 5 Malawi-Liverpool-Wellcome Trust Clinical Research Programme, Malawi; 6 Liverpool School of Tropical Medicine Department of Clinical Sciences, UK; 7 Department of Geomatic Engineering, School of Engineering, University of Zambia, Zambia

**Keywords:** analytical hierarchical process (AHP), GIS, malaria risk, Nsanje, sensitivity

## Abstract

Malaria remains one of the critical public health threats, particularly in endemic sub-Saharan countries like Malawi. Although malaria prevalence has declined over the years, the disease continues to pose a notable public health burden, contributing to high levels of morbidity and mortality. This study mapped malaria risk in Nsanje District, southern Malawi. Environmental variables (temperature, rainfall, elevation, slope, and proximity to rivers) were used to model malaria hazard, while socio-economic factors (proximity to health facilities and roads, and population density) defined vulnerability. Land use and land cover derived from Sentinel-2 using the random trees classifier in ArcGIS Pro were used to delineate elements at risk. The analytical hierarchy process and weighted overlay analysis were applied to generate hazard, vulnerability, and risk maps. Additionally, sensitivity analysis was conducted to determine the most influential factors. Results show that temperature and rainfall contributed more within the model to malaria hazard, with 20.4% and 35.1% of the area classified as high- and very high-hazard zones, respectively. Vulnerability was mainly affected by proximity to health facilities and population density, while 43.1% of the district was categorized as high risk and 40.5% as moderate risk. Overall, malaria hazard contributed most to the total risk, followed by vulnerability. The findings of this study are essential for understanding the complex dynamics of malaria transmission, which are influenced by a combination of environmental, climatic, and socio-economic factors. The study recommends enhancing healthcare accessibility and developing early warning systems, including malaria risk maps, to support targeted prevention and control efforts. Future studies should integrate long-term climate projections and real-time, high-frequency environmental and epidemiological data to enhance malaria risk modelling.

## Introduction

As one of the leading vector-borne diseases, malaria continues to impose a substantial global burden through widespread illness and preventable deaths. The cause of malaria is infection with Plasmodium parasites, which are spread through bites from female Anopheles mosquitoes. Four species infect humans: *Plasmodium falciparum, Plasmodium vivax, Plasmodium malariae*, and *Plasmodium ovale* with *P. falciparum* and *P. vivax* being the most predominant [1]. The worldwide relevance of malaria control is highlighted in the 2030 Agenda for Sustainable Development, which identifies the reduction of malaria incidence as a key target under Sustainable Development Goal 3 (SDGs) [2]. Malaria ranks as the sixth leading cause of mortality in low-income nations, predominantly located in Africa [3]. The World Health Organization indicated that around 249 million cases of malaria were reported in 85 malaria-endemic countries in 2022. Furthermore, over 608,000 fatalities were attributed to malaria worldwide, resulting in a mortality rate of 14.3 deaths per 100,000 individuals at risk [4]. These statistics highlight the life-threatening nature of the disease, particularly in subtropical and tropical regions.

In the sub-Saharan Africa region, malaria remains one of the most devastating public health challenges, where the region accounts for approximately 94% of global malaria cases and 95% of deaths, with an estimated 233 million cases and 580,000 deaths reported in 2022 [5]. In Malawi, the malaria prevalence had decreased to 10.5% by 2021, representing a reduction of approximately 52% compared to the 2016 prevalence. Despite the drop, Malawi remains among the top 20 countries with the highest malaria prevalence and mortality rates globally. According to 2023 estimates, the country accounted for approximately 1.8% of global malaria cases and 1.2% of global malaria deaths. Within the Eastern and Southern African region, about 8% of all malaria cases were reported in Malawi [1]. The disease peaks after the annual rains, which generally start in November or December and continue until April [6].

Lowland regions with high temperatures and low altitudes, such as the Lower Shire Valley, provide ideal conditions for malaria transmission [6]. Within this valley, Nsanje District exhibits one of the highest malaria prevalence rates in Malawi, estimated at approximately 42% among children under 5 years of age [7]. Nsanje District was selected as the study area because it represents a uniquely high-risk ecological and socio-spatial setting within the Lower Shire Valley. The district is characterized by persistently high temperatures, low elevation, proximity to the Shire River system, and seasonal flooding, all of which favour vector breeding and malaria transmission. In addition, Nsanje has a largely rural settlement structure, where distance-based barriers to health services remain significant, making it an appropriate district for evaluating vulnerability related to healthcare accessibility. Although other districts within the valley also experience high malaria prevalence, Nsanje provides a critical case study for district-scale risk mapping due to its documented disease burden, environmental suitability, and operational need for spatial targeting of interventions. While notable progress has been made in malaria control interventions in Malawi since 2022 [8], recent district-level evidence indicates that malaria vaccine uptake in Nsanje remains below recommended targets [9]. Vaccine uptake is not directly included as a spatial criterion in the current GIS–AHP modelling framework; however, this evidence reinforces the need for complementary spatial decision-support tools, such as malaria risk maps, to guide targeted prevention and resource allocation.

The virulence of malaria is exacerbated by several factors, including the resistance of *Anopheles spp.* to control mechanisms, climate change, limited global funding, weaknesses in health systems, and changes in human immune systems [10]. Environmental factors, such as temperature, the presence of water, and altitude, lead to modifications in the behaviour and spatial distribution of malaria vectors [11]. Besides environmental factors, socio-economic factors such as population density and the accessibility of healthcare services define the vulnerability of the population in high-risk areas. Increased distance from health facilities exacerbates population vulnerability by imposing economic burdens, delaying diagnosis and treatment, and discouraging healthcare-seeking behaviour [12, 13]. These barriers can lead to worse individual clinical outcomes and help sustain community-level transmission.

Geographical Information Systems (GIS) are invaluable for mapping and studying the spread of mosquito-borne diseases, such as malaria, which helps identify areas at high risk for focused interventions [14]. GIS, combined with Remote Sensing, has been applied to model and understand the temporal and spatial variations of environmental variables and their relationships to disease vectors [15]. AHP-based GIS approaches have been widely applied in health geography and disease risk mapping due to their transparency, ease of interpretation, and ability to integrate expert knowledge with spatial data [16, 17]. While these methods do not provide statistical inference or uncertainty quantification in the manner of model-based geostatistics or spatial regression, they offer a pragmatic framework for synthesizing multiple data layers and stakeholder perspectives to guide resource allocation and intervention planning [18, 19].

Recent studies in Malawi have explored malaria risk and control measures; however, most have concentrated on national or regional scales, leaving district-level assessments, such as those in Nsanje, relatively unexamined. There is a lack of comprehensive malaria risk mapping that integrates environmental, meteorological, and socio-economic factors. Research by [20] utilized spatial regression models to analyse malaria risk in northern Malawi, highlighting the influence of environmental variables such as temperature, rainfall, and altitude on malaria distribution. Additionally, Ahmed [19] showcased GIS-based platforms for mapping and hotspot identification in southern Malawi. Meanwhile, Zolekar and Bhagat [18] investigated malaria trends among children under five from 2015 to 2019 and developed a model to predict cases from 2020 to 2022. This study employs a GIS-based multi-criteria evaluation framework combining the analytical hierarchy process (AHP) with weighted overlay analysis. This approach was selected for its practical applicability in resource-limited settings where data availability and technical capacity may constrain the use of more complex statistical models [21, 22]. The primary objective is to develop an accessible, interpretable risk mapping tool that can inform targeted intervention strategies rather than to conduct formal epidemiological inference or establish causal relationships between risk factors and malaria transmission in Nsanje District. The study supports UN SDG No. 3 and AU Agenda 2063 Goal No. 3, contributing to improved risk mapping and surveillance for targeted resource prioritization in malaria eradication efforts.

## Materials and methods

### Study area

**Figure 1.**
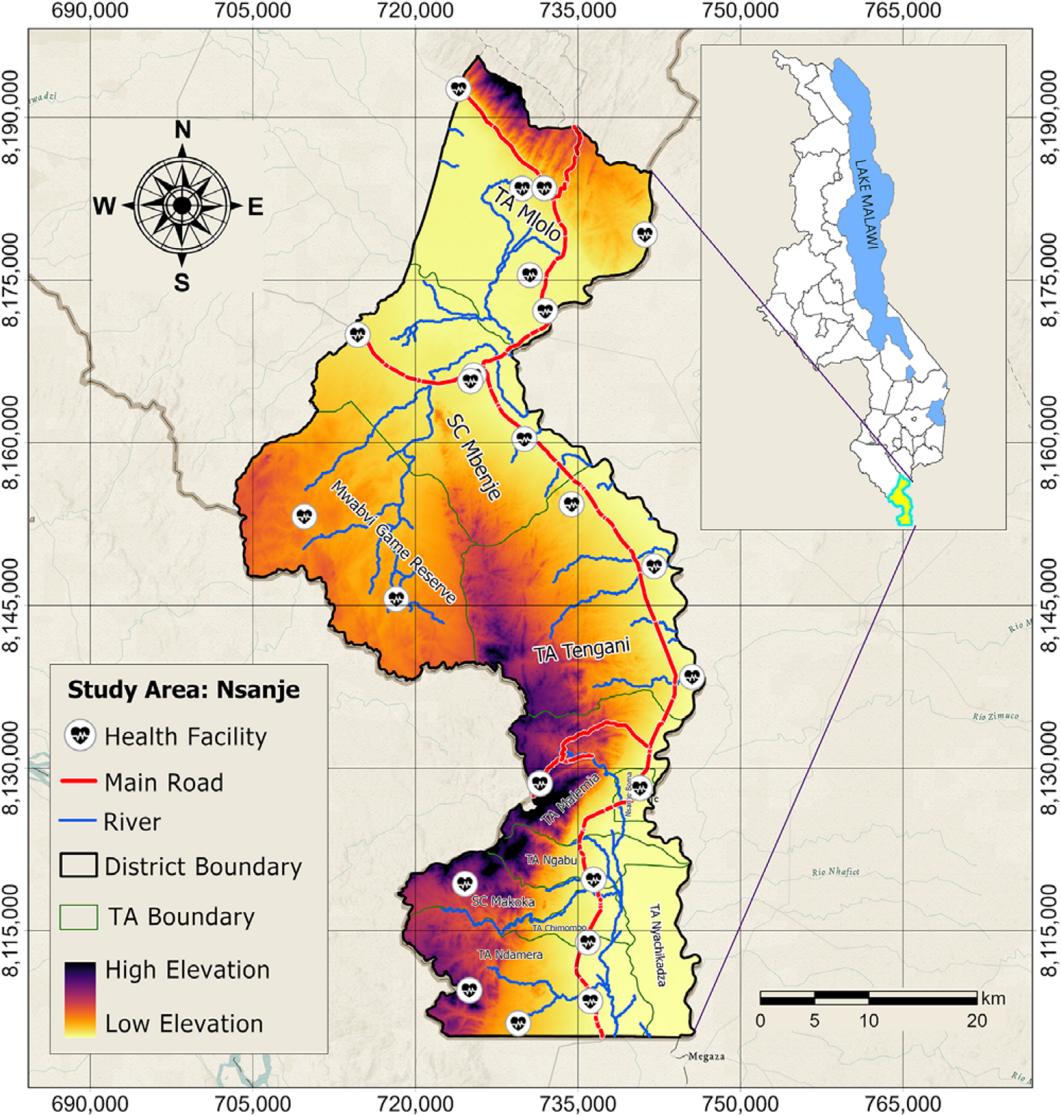
Location of the study area. Figure 1. long description.

The study was conducted in Nsanje District, located in the Lower Shire Valley of southern Malawi (Figure 1). The district is located at 16.7288° S, 35.1709° E, with altitudes ranging from 28 m to 961 m above MSL. The district has a total of 11 Traditional Authority (TA) divisions, with 24 health facilities serving at least 300,000 individuals [23]. Average annual temperatures in Nsanje range from 19°C to 36°C, rarely falling below 17°C or climbing above 41°C [24].

### Data collection

A total of five environmental and physical factors were considered in this study for hazard analysis. These are atmospheric temperature, rainfall, elevation, slope, and proximity to rivers. The analysis was conducted in ArcGIS Pro. The datasets and sources are summarized in Table 1.

**Table 1.** Data used for the study, their functions, and sources Table 1. long description.

Data	Resolution	Purpose	Source
Temperature (Jan–Dec 2024)	0.5° × 0.625°	Temperature map	*NASA POWER*
Rainfall	0.05°	Rainfall map	CHIRPS
SRTM DEM	30 m	Elevation, slope, and river proximity maps	*USGS*
Roads	–	Proximity to roads	*OpenStreetMap*
Health facilities	–	Proximity to health facilities	MoH
Population density		Population density map	*NSO*
Sentinel–2 imagery	10-m	LULC map	Copernicus (ESA)
Malaria incidence	–	Malaria risk comparison	DHIS2
Administration boundaries	–	Study area, country, TA boundaries	*DIVA GIS*

**Figure 2.**
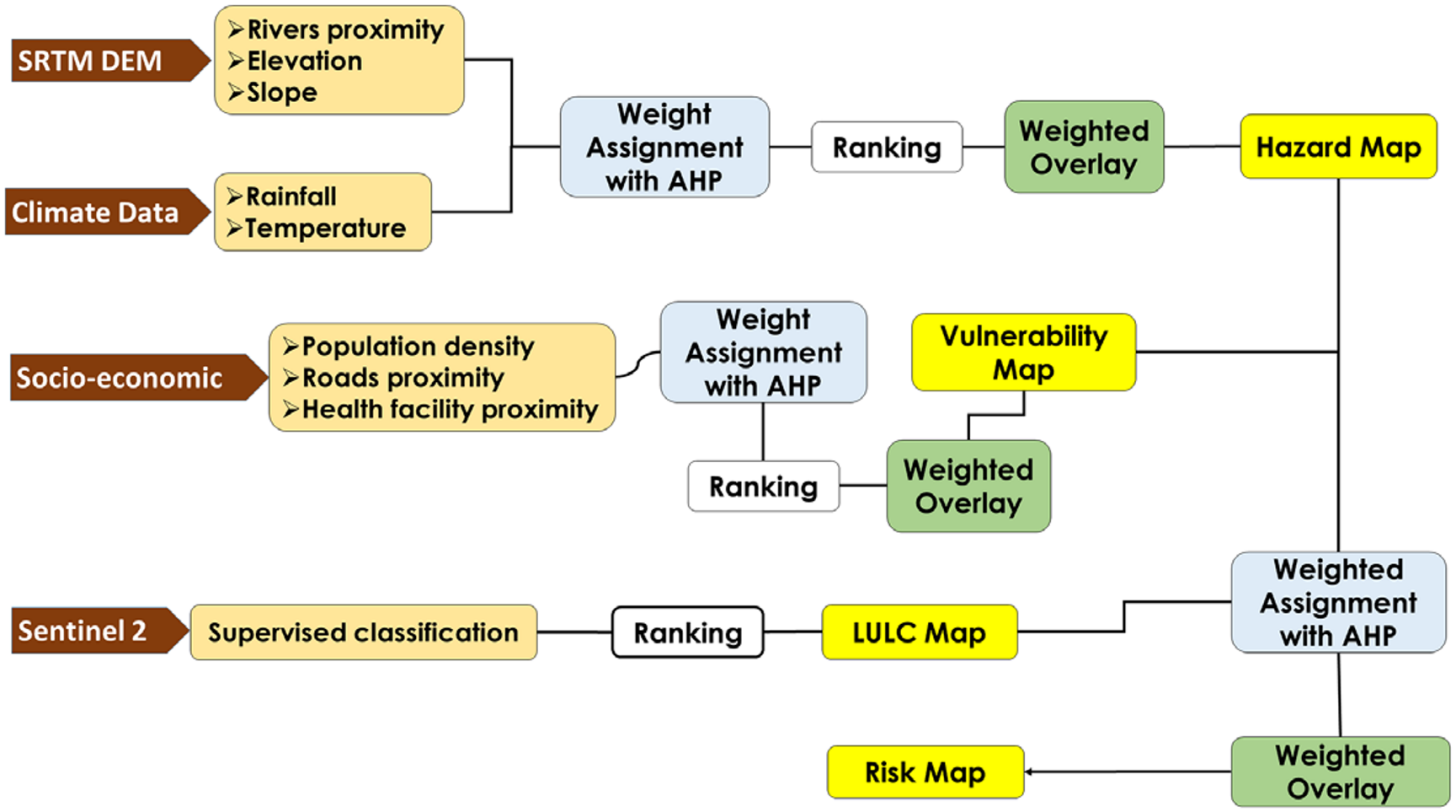
Methodology workflow for the study. Figure 2. long description.

The methodological workflow adopted for the study is presented in Figure 2.

### Data analysis

#### Weight calculation for contributing factors

The AHP was employed to assign weights. A pairwise comparison matrix (PCM) was separately created for each malaria hazard, vulnerability, and risk factor [25, 26]. Scores, ranging from 1 to 9, were assigned to each criterion based on expert judgements and a literature review [27–29]. The matrix was then used to compute relative weights for all the factors involved in each analysis. A consistency ratio (CR) is used to assess the reliability of the weights. Weights are acceptable if CR is less than 10%, and must be revised if greater than 10% [30]. CR was calculated using equation (1):

(1) $$CR=\frac{CI}{RI}<0.1$$

where CI is the consistency index, which evaluates logical inconsistencies in experts’ judgements in the PCM, and RI is a random index, averaging the CI of a large set of randomly generated matrices of size 𝑛 [31]. CI was calculated using equation (2):

(2) $$CI=\frac{\left(\lambda_{m}-n\right)}{\left(n-1\right)}$$

where $\lambda_{m}$ represents the principal eigenvalue of the PCM and n indicates the order of the matrix.

#### Hazard assessment

**Figure 3.**
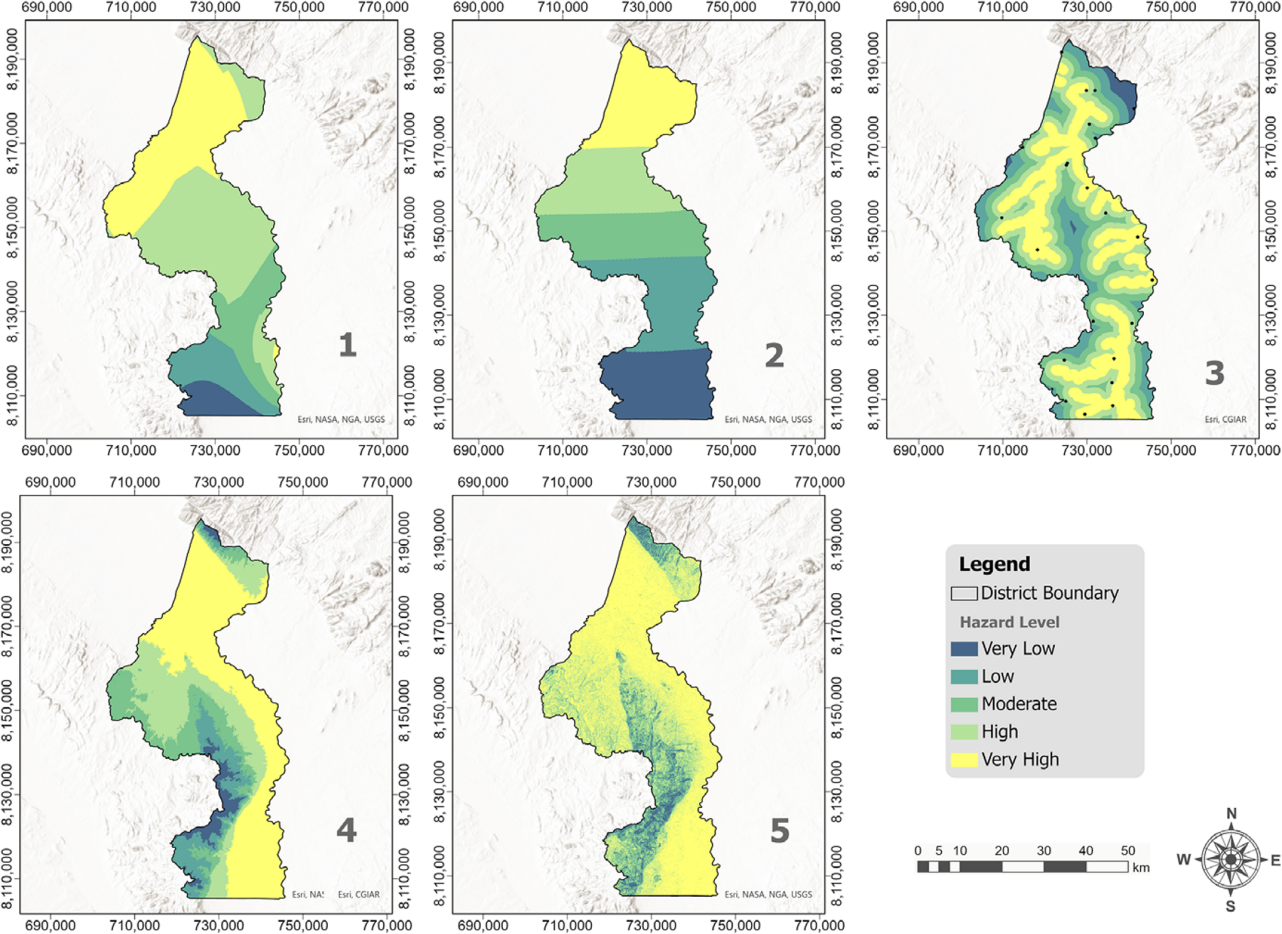
Visual representation of the classification of the malaria hazard factors; Temperature (1), Rainfall (2), Proximity to river (3), Elevation (4), and Slope (5) in Nsanje District, Southern Malawi. Figure 3. long description.

Hazard refers to the possibility that a potentially dangerous occurrence, such as the spread of malaria, will take place within a given time frame and geographical area. In this study, the term hazard is used in its technical sense within spatial epidemiology and disaster-risk modelling, referring to the environmental suitability for malaria transmission. Hazard therefore represents the likelihood that climatic and physical conditions support vector breeding and parasite development, independent of population exposure or access to health services. To integrate heterogeneous environmental datasets into the AHP–weighted overlay framework, continuous variables were standardized into ordinal suitability strata (very low to very high) following established GIS multi-criteria evaluation procedures [32]. The five environmental and physical variables (temperature, rainfall, elevation, slope, and proximity to rivers) (Figure 3) were first classified into five classes each using the Natural Breaks (Jenks) classification method in ArcGIS Pro. Rainfall was classified using a non-linear suitability assumption, recognizing that malaria transmission does not necessarily increase monotonically with rainfall. While moderate rainfall creates stagnant pools favourable for breeding, very high rainfall may reduce vector suitability through flushing of larval habitats and increased flow velocities, particularly in lowland flood-prone settings [7, 33]. Therefore, the highest rainfall class was assigned lower hazard suitability, consistent with evidence that excessive rainfall can suppress larval survival and disrupt breeding habitats. Table 2 shows class values ranging from 1 (very low) to 5 (very high).

**Table 2.** Classifications of malaria hazard contributing factors Table 2. long description.

Factor	Class	Rank	Hazard
Rainfall (mm)	1,490–1871	1	Very Low
	1,117–1,490	2	Low
	743–1,117	3	Moderate
	376–743	4	High
	3–376	5	Very High
Temperature ( ${}^{\circ }C$ )	19–21	1	Very Low
	21–22	2	Low
	22–23	3	Moderate
	23–24	4	High
	24–25	5	Very High
Slope (degree)	21–60	1	Very Low
	12–21	2	Low
	6–12	3	Moderate
	1–6	4	High
	0–1	5	Very High
Elevation (m)	507–961	1	Very Low
	331–507	2	Low
	203–331	3	Moderate
	104–203	4	High
	28–104	5	Very High
Proximity to Rivers (m)	6,008–11,265	1	Very Low
	3,799–6,008	2	Low
	2,121–3,799	3	Moderate
	707–2,121	4	High
	0–707	5	Very High

#### Vulnerability assessment

**Figure 4.**
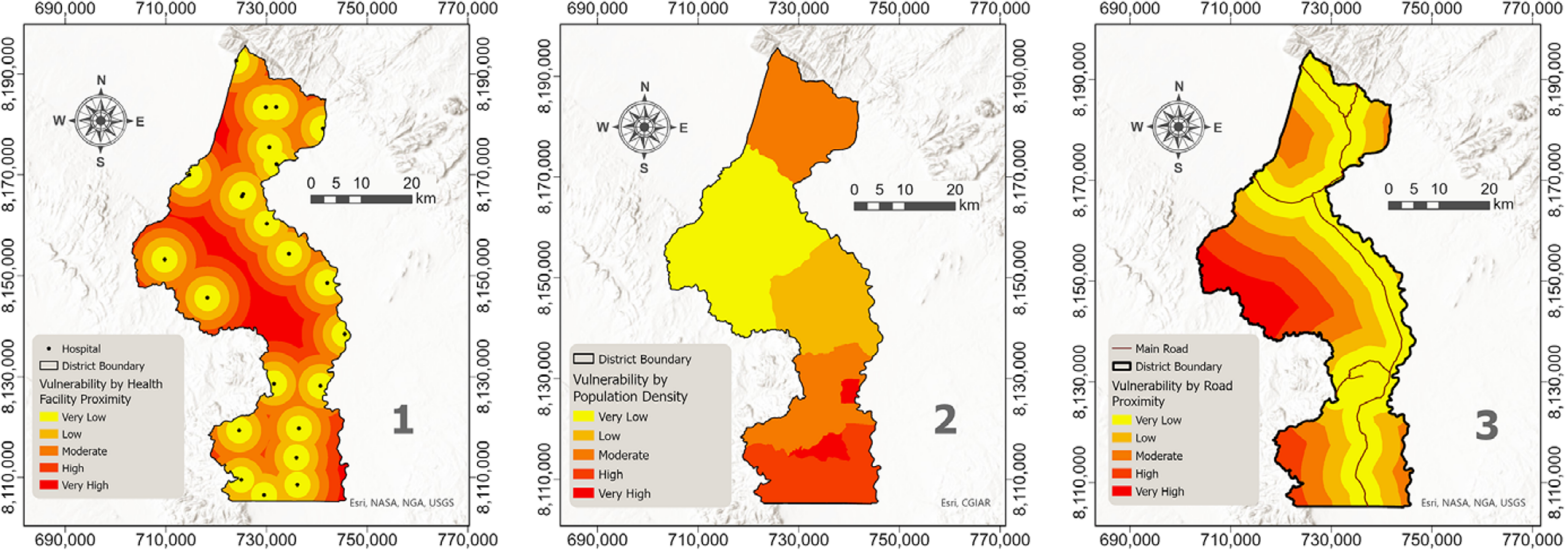
Visual representation of the classification of malaria vulnerability factors: proximity to health facilities (1), population density (2), and proximity to roads (3) in Nsanje District, Southern Malawi. Figure 4. long description.

Vulnerability refers to the susceptibility of particular elements or groups to the negative impacts of malaria, which vary in severity. Each of the vulnerability factors (Table 3), such as proximity to health facilities, population density, and proximity to roads, was classified into five classes (very high to very low) using the Natural Breaks (Jenks) method in ArcGIS Pro. Figure 4 presents a visual representation of the classification of malaria vulnerability factors. Population density was included as a vulnerability proxy representing the spatial concentration of potentially exposed individuals. It is acknowledged that in highly urbanized contexts, higher population density may correlate with lower malaria risk due to improved housing, infrastructure, and healthcare access. However, in predominantly rural districts such as Nsanje, higher population density typically reflects clustered settlements where human–vector contact may be intensified, while health-system capacity remains limited. For this reason, higher population density was ranked as higher vulnerability in this district-level context.

**Table 3.** Classifications of malaria vulnerability factors Table 3. long description.

Factor	Class	Rank	Vulnerability
Population Density (persons/km^2^)	25–40	1	Very Low
	40–64	2	Low
	64–169	3	Moderate
	169–501	4	High
	501–1741	5	Very High
Proximity to Health Facility (m)	0–1,287	1	Very Low
	1,287–3,552	2	Low
	3,552–5,612	3	Moderate
	5,612–8,031	4	High
	8,031–13,128	5	Very High
Proximity to Roads (m)	0–1,694	1	Very Low
	1,694–5,271	2	Low
	5,271–9,789	3	Moderate
	9,789–15,248	4	High
	15,248–24,002	5	Very High

#### Land use and land cover assessment

In this study, elements at risk (E) refer to land-based features that represent the spatial distribution of human presence, activities, and ecological conditions that influence exposure to malaria hazard. Land use and land cover (LULC) therefore provides a proxy for where people live (settlements), where they work (farmland), and where mosquito breeding habitats are concentrated (water bodies). By integrating LULC into the risk model, we account for the spatial distribution of exposed environments that mediate contact between humans and vectors. LULC are crucial indicators for assets on the land that influence malaria transmission [34]. A supervised classification using the Random Trees classifier in ArcGIS Pro was performed on the 2024 Sentinel 2 satellite imagery, considering five LULC classes, namely, vegetation, water, settlement, bare/grassland, and farmland (Table 4). Water bodies were assigned the highest value (5), while vegetation was assigned the lowest value (1) [27, 35].

**Table 4.** Categorization of elements at risk Table 4. long description.

Element at Risk	Rank	Susceptibility
Vegetation	1	Very Low
Bare/Grassland	2	Low
Farmland	3	Moderate
Settlement	4	High
Water	5	Very High

#### Risk assessment

Risk is defined as a potentially damaging event that may arise, threatening the well-being of a society. It is the probability that a specific disease infects individuals over a specific timeframe [19]. The model formula (Equation 3) for calculating risk considers hazard, vulnerability, and elements at risk to assess risk levels across regions [19]. The final malaria risk surface was generated using a two-stage integration process. First, hazard and vulnerability were each modelled as composite indices using AHP-derived weights applied to their respective standardized criteria layers (hazard: temperature, rainfall, elevation, slope, and river proximity; vulnerability: population density, proximity to health facilities, and proximity to roads). Second, elements at risk were derived from the Sentinel-2 LULC classification and reclassified into ordinal susceptibility ranks. Finally, the three composite layers (hazard, vulnerability, and elements at risk) were integrated using AHP-derived weights to generate the final malaria risk map. No variables were excluded; all criteria were retained within their respective components. The malaria risk map was generated using a weighted linear combination of the three composite components (hazard, vulnerability, and elements at risk), following GIS multi-criteria evaluation procedures. Risk was computed as:

(3) Risk=(wH×H)+(wV×V)+(wE×E)

where H is the hazard index; V is the vulnerability index; E represents elements at risk (LULC susceptibility); and wH, wV, and wE are the AHP-derived weights assigned to each component. The weight for each input map was calculated using the AHP, using expert judgements and several literature reviews [28, 29]. Finally, the risk map was overlaid with cumulative malaria incidence from 2014 to 2024 for comparison. The resulting continuous hazard, vulnerability, and risk indices were reclassified into five ordinal categories (very low, low, moderate, high, and very high) using the Natural Breaks (Jenks) classification method in ArcGIS Pro to support interpretability and decision-making.

#### Sensitivity of factors in malaria risk mapping

The sensitivity of the hazard, vulnerability, and overall risk components was assessed to determine how variations in the criterion weights affected the model outputs. Here, the term risk components refer to the three composite spatial layers (hazard, vulnerability, and elements at risk) that are integrated to generate the final malaria risk surface. The weighting scheme proposed by [36] was adopted, in which one criterion layer was assigned weights of 10%, 30%, 50%, and 70% in weighted overlay analyses, while the remaining criterion layers were assigned equal weights in each iteration (Table 5). The number of rows, except headers, indicates the number of iterations each model was run. The sensitivity analysis was conducted for hazard and vulnerability criteria because these components are constructed from multiple independent variables. The elements-at-risk component (LULC) was derived from a single classified dataset and therefore treated as one composite layer rather than multiple criteria layers. Consequently, Table 5 presents sensitivity iterations for hazard criteria, vulnerability criteria, and the three integrated risk components (hazard, vulnerability, and elements at risk).

**Table 5.** Weighting scheme for hazard criteria, vulnerability criteria, and risk factors Table 5. long description.

Hazard criteria					
Model run	Temperature (%)	Rainfall (%)	Slope (%)	Elevation (%)	River proximity (%)
1	10	22.5	22.5	22.5	22.5
2	30	17.5	17.5	17.5	17.5
3	50	12.5	12.5	12.5	12.5
4	70	7.5	7.5	7.5	7.5
5	22.5	10	22.5	22.5	22.5
6	17.5	30	17.5	17.5	17.5
7	12.5	50	12.5	12.5	12.5
8	7.5	70	7.5	7.5	7.5
9	22.5	22.5	10	22.5	22.5
10	17.5	17.5	30	17.5	17.5
11	12.5	12.5	50	12.5	12.5
12	7.5	7.5	70	7.5	7.5
13	22.5	22.5	22.5	10	22.5
14	17.5	17.5	17.5	30	17.5
15	12.5	12.5	12.5	50	12.5
16	7.5	7.5	7.5	70	7.5
17	22.5	22.5	22.5	22.5	10
18	17.5	17.5	17.5	17.5	30
19	12.5	12.5	12.5	12.5	50
20	7.5	7.5	7.5	7.5	70

Vulnerability criteria			
Model run	Population density (%)	Hospital proximity (%)	Road proximity (%)
1	10	45	45
2	30	35	35
3	50	25	25
4	70	15	15
5	45	10	45
6	35	30	35
7	25	50	25
8	15	70	15
9	45	45	10
10	35	35	30
11	25	25	50
12	15	15	70

Risk factors			
Model run	Hazard (%)	Vulnerability (%)	Elements at risk (%)
1	10	45	45
2	30	35	35
3	50	25	25
4	70	15	15
5	45	10	45
6	35	30	35
7	25	50	25
8	15	70	15
9	45	45	10
10	35	35	30
11	25	25	50
12	15	15	70

## Results

### Malaria hazard map of Nsanje District

**Figure 5.**
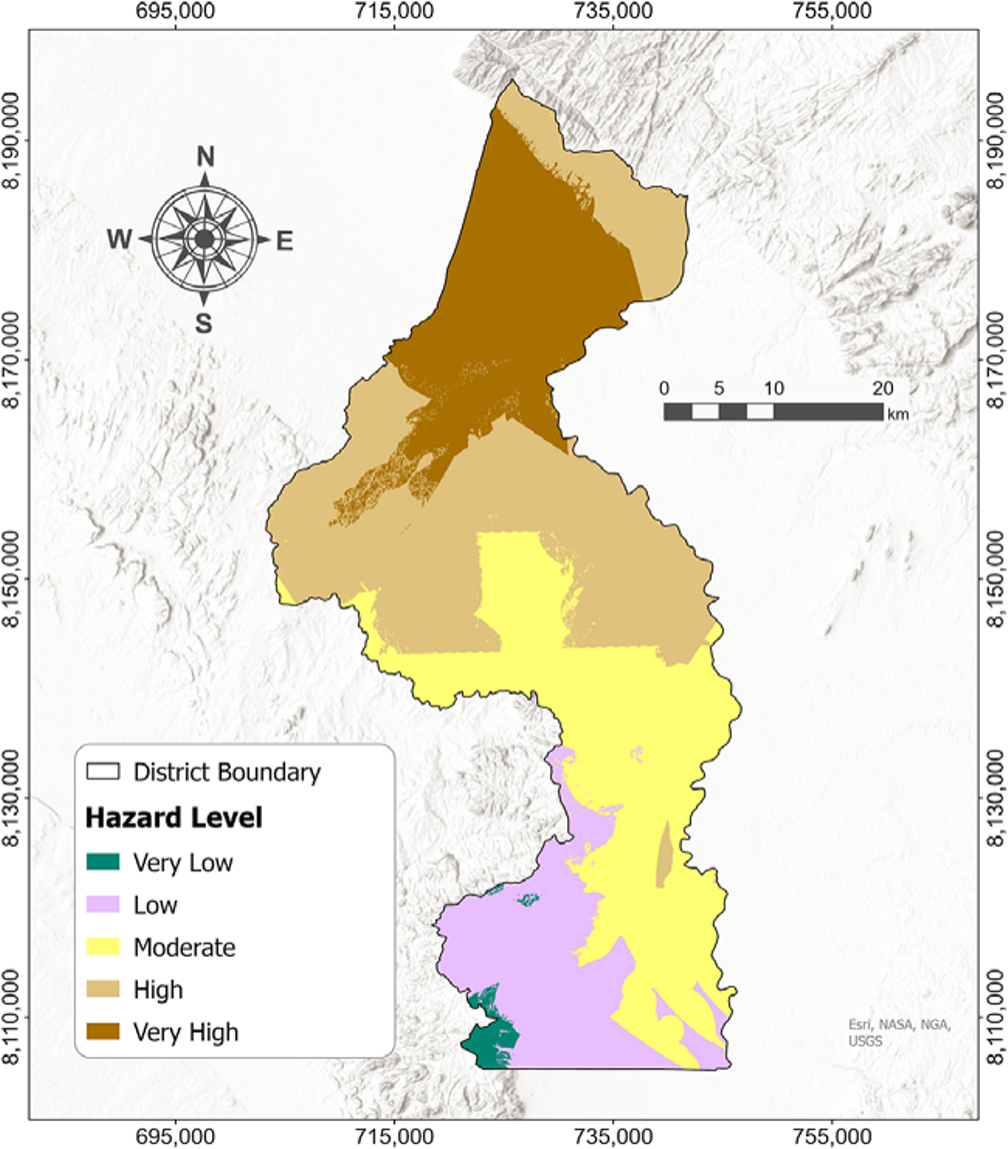
Spatial distribution of the five classes of malaria hazard in Nsanje District. Figure 5. long description.

The regions with a high malaria hazard cover an area of 679.8 km^2^, accounting for approximately 35.1% of the study area. The very high hazard zones cover 20.4% of the study area, with only 20.3 km^2^ (1% of the study area) in the very low hazard zone. Figure 5 shows the area coverage of each hazard class. The relative weights for the hazard factors used in the overlay are presented in Table 6, as calculated through the AHP, with an acceptable CR of 4.3%. Temperature and rainfall, with equal weights of 34.9%, had higher weighting in malaria hazard.

**Table 6.** Relative weights for malaria hazard factors Table 6. long description.

Factor	Weight (%)
Rainfall	34.9
Temperature	34.9
Slope	5.1
Elevation	10.7
Proximity to Rivers	14.3

### Malaria vulnerability map of Nsanje District

**Figure 6.**
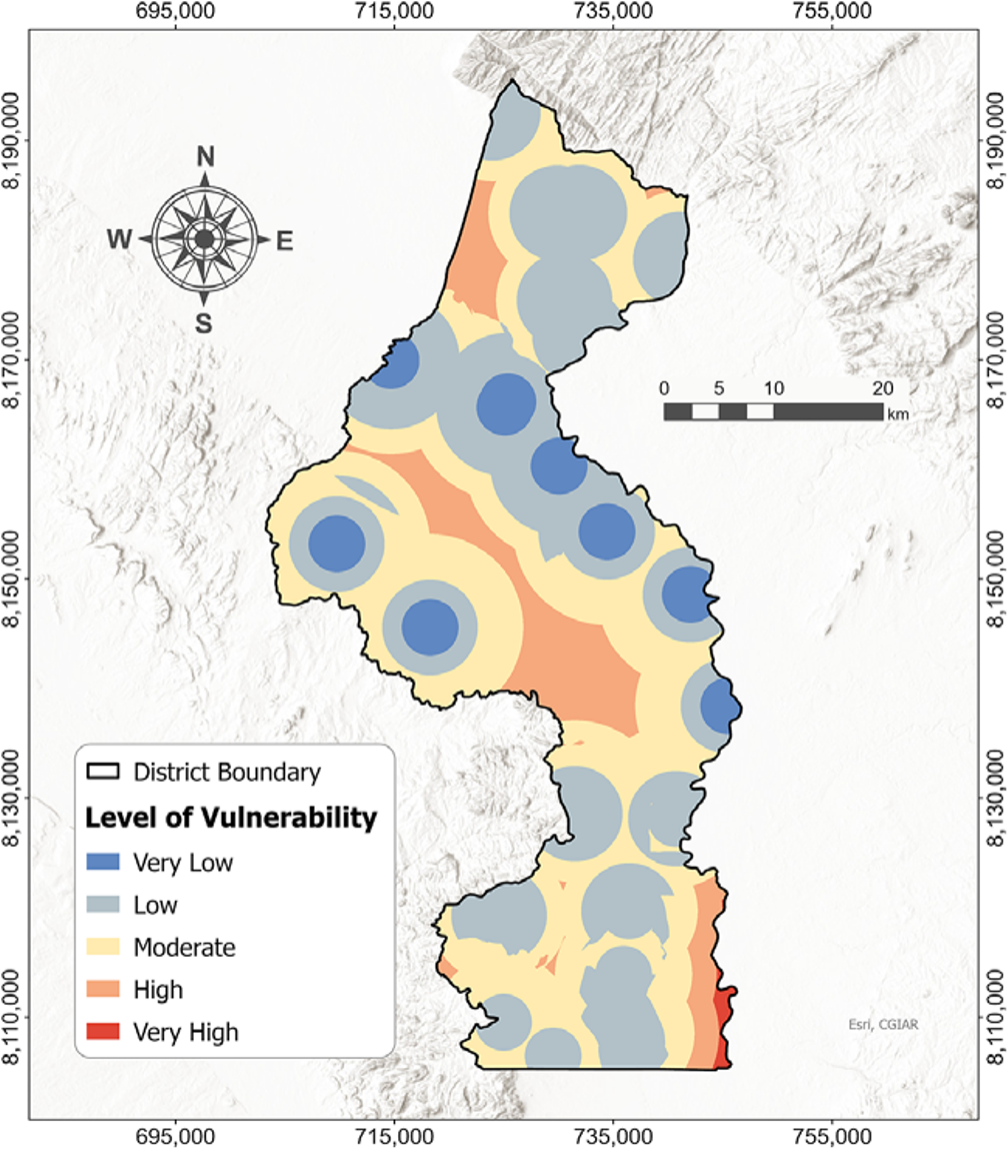
Distribution of malaria vulnerability in Nsanje District. Figure 6. long description.

The low vulnerability class covers 41.6% (807.6 km^2^) of the study area, followed by the moderate class, which covers 38.2% (740.4 km^2^) of the study area, as displayed in Figure 6. The very high vulnerability class has the smallest area, 8.6 km2, or 0.4% of the study area. The weight calculation revealed that Proximity to Health Facilities has the highest contribution to malaria vulnerability, with a weight of 63.7%, with, Proximity to roads contributing the least. The AHP model used achieved a CR of 4%, indicating consistency. Table 7 summarizes the weights for the vulnerability factors.

**Table 7.** Relative weights for malaria vulnerability factors Table 7. long description.

Factor	Weight (%)
Population density	25.8
Health facility proximity	63.7
Road proximity	10.5

### Elements at risk map of Nsanje District

**Figure 7.**
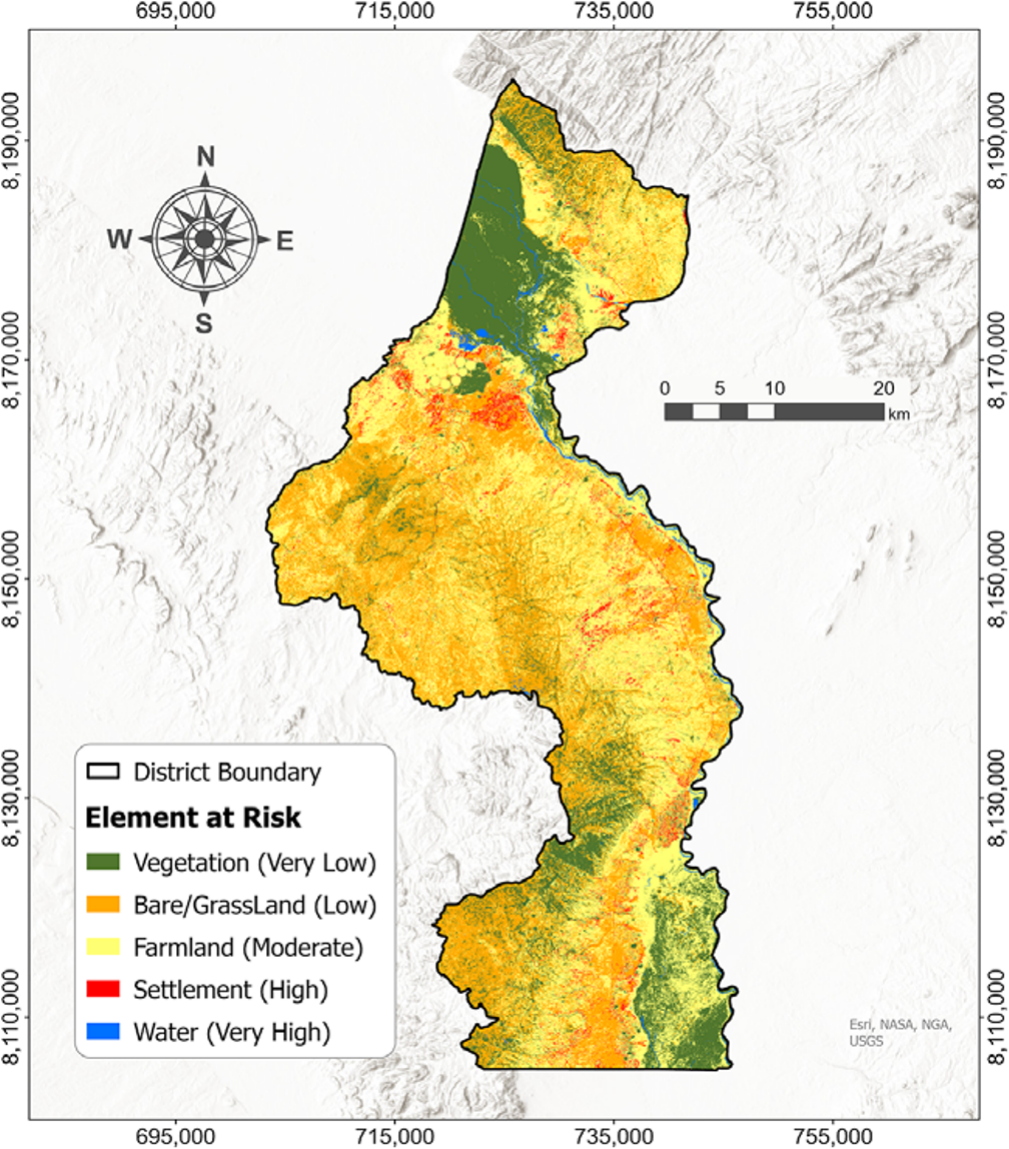
2024 land use and land cover map of Nsanje District, displaying ranked elements at risk. Figure 7. long description.

The map in Figure 7 shows the LULC classifications of the study area and their respective levels of malaria risk. Farmland occupies the most area, at 847.6 km^2^, accounting for approximately 43.7% of the study area. Bare/Grassland is the second-most covering area (675.9 km2), followed by water bodies, which cover the least area (0.8% of the study area). The areal coverage for each LULC class is presented in Table 8.

**Table 8.** Areal coverage for LULC classes Table 8. long description.

Element at risk	**Area (km** ^**2**^**)**	Percentage (%)
Vegetation	341.5	17.6
Bare/Grassland	675.9	34.9
Farmland	847.6	43.7
Settlement	57.9	3.0
Water	16.2	0.8

### Malaria risk map of Nsanje District

**Figure 8.**
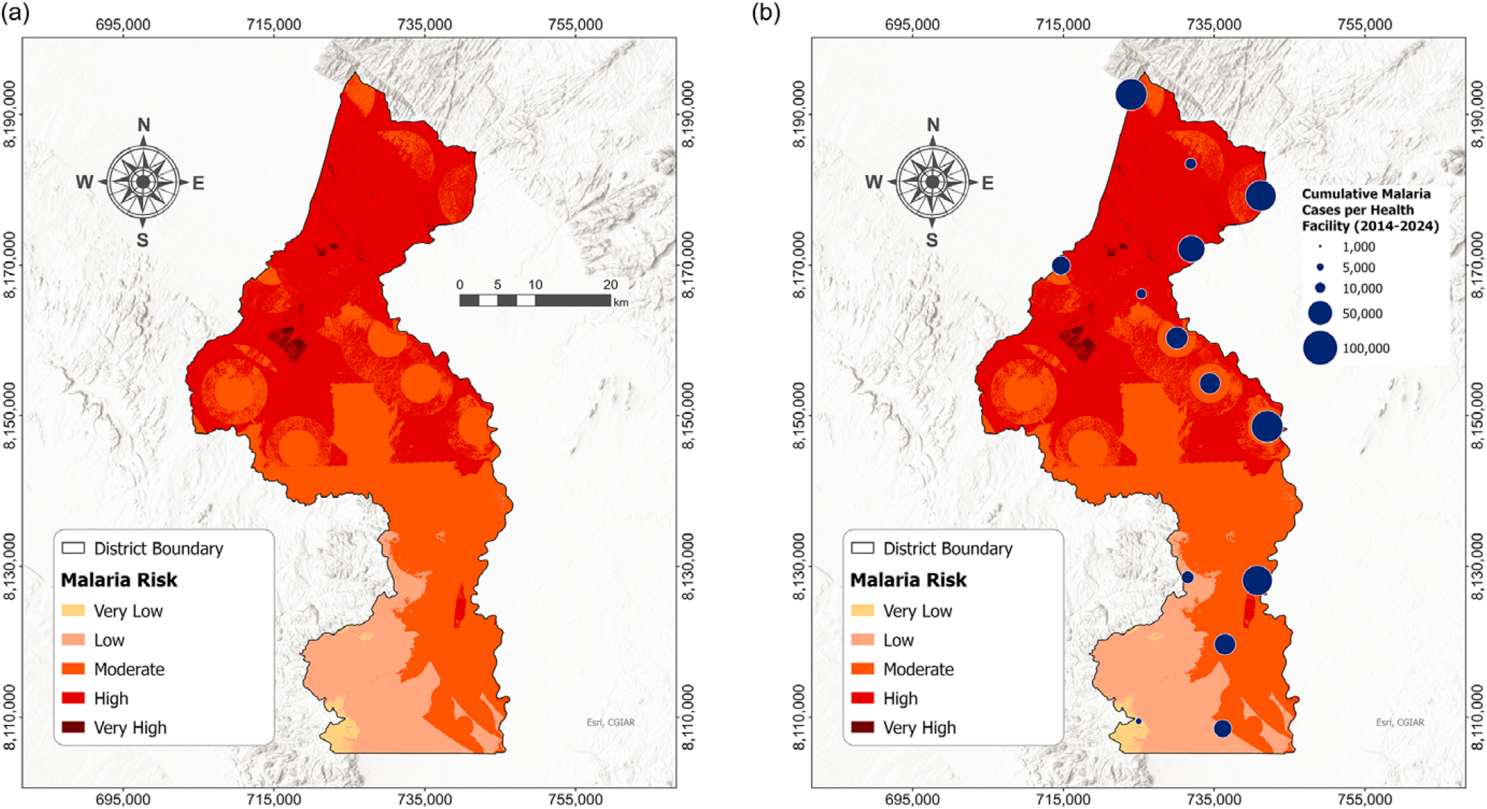
(a) Malaria risk map. (b) Malaria risk and incidence comparison map. Figure 8. long description.

The malaria risk across the study area is shown in Figure 8(a). The high-risk class dominates the study area, covering 835.8 km^2^, or 43.1% of the study area. However, the smallest area, 13.4 km^2^ (0.7%), is covered by the very high-risk class, followed by the very low-risk class at approximately 19.4 km^2^. The model achieved an acceptable CR of 6.8%, with the hazard criteria, vulnerability criteria, and elements at risk accounting for 73.1%, 18.8%, and 8.1%, respectively, in determining the risk zone. Figure 8(b) shows that most facilities with the highest number of malaria incidence are located within the high-risk zones.

### Criteria sensitivity

**Figure 9.**
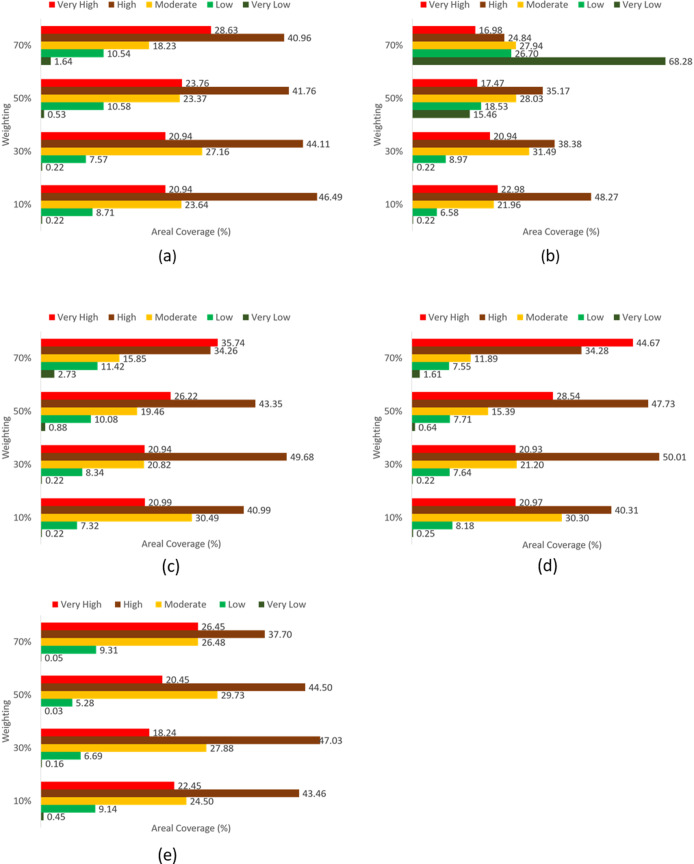
Sensitivity to malaria hazard of (a) temperature, (b) rainfall, (c) elevation, (d) slope, and (e) river proximity. Figure 9. long description.

**Figure 10.**
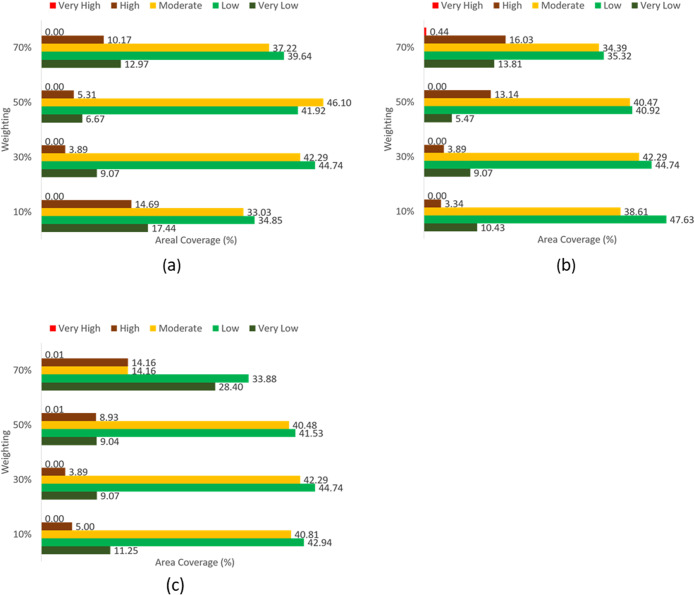
Sensitivity to malaria vulnerability of (a) population density, (b) proximity to health facility, and (c) proximity to roads. Figure 10. long description.

**Figure 11.**
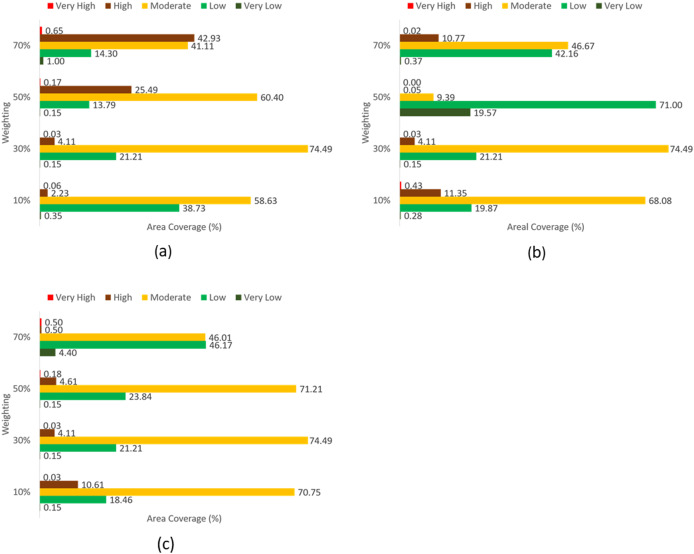
Sensitivity to malaria risk of (a) hazard, (b) vulnerability, and (c) elements at risk. Figure 11. long description.

Figure 9 illustrates the sensitivity of malaria hazard factors across four weighting schemes (10%, 30%, 50%, and 70%). Notable variations in areal coverage are seen for temperature (Figure 9a) and rainfall (Figure 9b), while elevation (Figure 9c) shows modest changes, and slope (Figure 9d), along with proximity to rivers (Figure 9e), exhibit lesser sensitivity. Figure 10 shows the areal dynamics across vulnerability classes, indicating that proximity to health facilities (Figure 10(a)) is highly sensitive, notably expanding high-vulnerability areas with increased weighting. Population density (Figure 10(b)) shows moderate variations, and proximity to roads (Figure 10(c)) is the least sensitive factor. Figure 11(a) hazard, (b) vulnerability, and (c) elements at risk reveal that malaria hazard factors are most sensitive to changes in weight, particularly affecting the high-risk class. In contrast, malaria vulnerability is moderately sensitive, and elements at risk display the least sensitivity.

## Discussion

The present study demonstrates that climatic factors, particularly temperature and rainfall, exert the most decisive influence on malaria hazard in Nsanje District. AHP results identified these variables as equally dominant contributors, a finding which closely aligns with earlier research emphasizing the centrality of temperature and rainfall in shaping malaria transmission potential across Africa [27–29]. The dominant influence of temperature is supported by its well-established biological role in accelerating the development rates of both the Anopheles mosquito vector and the Plasmodium parasite. Given that optimal transmission occurs between 20 °C and 30 °C [37, 38], Nsanje’s observed temperature range (19 °C–25 °C) constitutes a persistently favourable environment for malaria transmission. The sensitivity analysis further reinforced this relationship by revealing substantial shifts in the spatial distribution of high-hazard areas when the weights for these variables were increased. This indicates that malaria hazard patterns in the district are highly responsive to climatic variability. However, it should be noted that all the sensitivity analysis results only reflect model sensitivity, and not causal importance. The obtained results indicate how assumptions affect model outputs, not empirical determinants of malaria risk.

Rainfall was also identified as a dominant hazard driver in the AHP weighting scheme, receiving an equal weight to temperature (34.9%). This highlights that malaria hazard in Nsanje is not only shaped by thermal suitability but also by the hydrological conditions that govern the availability and stability of breeding habitats. Importantly, the relationship between rainfall and malaria hazard is often non-linear. While moderate rainfall typically increases hazard by creating shallow, stagnant pools suitable for Anopheles breeding, very high rainfall may reduce hazard by flushing larval habitats, increasing turbulence, and destabilizing breeding sites, particularly in flood-prone lowland systems. In Nsanje, which experiences seasonal flooding and riverine dynamics, excessive rainfall may therefore suppress larval persistence in certain areas, whereas areas with lower rainfall may still maintain breeding through permanent water bodies, residual pools, and river proximity. This explains why lower rainfall classes were ranked as higher hazard in the suitability reclassification, while rainfall remained an important driver overall in the integrated hazard model.

However, the current results diverge from the findings of Diriba et al. [39], who reported rainfall as the least influential factor and elevation as the strongest determinant of malaria hazard in Ethiopia. In Nsanje, a low-lying district characterized by consistently high temperatures and moderate rainfall seasonality, climatic conditions create favourable and relatively stable environments for Anopheles breeding. Consequently, elevation played a comparatively lesser role in shaping hazard patterns. Furthermore, despite the importance of river networks as breeding habitats, proximity to rivers exhibited weaker sensitivity than anticipated.

The vulnerability analysis revealed that accessibility to health facilities demonstrated higher influence for the population susceptibility to malaria. Areas located furthest from health facilities exhibited the highest vulnerability, underscoring the persistent challenge of geographic inaccessibility in malaria control. The measured distance to health facilities serves as a proxy for a more fundamental barrier to case management [13]. Greater travel distances exacerbate economic burdens and prolong delays in diagnosis and treatment, consequently increasing the risk of progression to severe disease and secondary transmission [12]. Therefore, the vulnerability mapped in this study embodies a population’s elevated risk of adverse health outcomes, stemming from systemic limitations in healthcare access [40]. These findings differ from those of [39], who identified population density as the principal determinant of vulnerability. Such divergence again emphasizes contextual differences. In Nsanje District, dispersed rural settlements and infrastructural constraints mean that physical access to diagnosis and treatment plays a greater role in shaping malaria vulnerability than population concentration alone. Proximity to roads showed limited influence, consistent with [34], who reported that road networks may play only an indirect role in shaping malaria risk unless they significantly affect healthcare accessibility. Although population density was included as a vulnerability proxy, its influence on the final malaria risk profile was comparatively weaker. This is partly explained by the modelling structure: vulnerability contributed less to overall risk than hazard, and within the vulnerability component, proximity to health facilities carried the dominant weight. Consequently, some high population density areas may still appear as low-to-moderate risk zones if they occur in environmentally less suitable locations (lower hazard) or closer to health services. This finding reinforces that in Nsanje, malaria risk is primarily governed by ecological suitability and healthcare accessibility rather than population concentration alone.

The assessment of LULC provided further insights into the distribution of elements at risk. Farmland was the dominant land cover type and was classified as moderately susceptible to malaria risk. Settlements and water bodies, which represent high and very high susceptibility categories, constituted relatively small proportions of the district. This finding aligns with [35], who similarly observed that rural, agriculturally dominated landscapes tend to exhibit moderate risk profiles because they lack large urban settlements but contain dispersed communities vulnerable to vector exposure. The extensive farmland coverage in Nsanje indicates a considerable population at risk of occupational exposure. Agricultural labourers working during dawn and dusk peak biting times for Anopheles mosquitoes are often unprotected by core interventions like insecticide-treated nets, which are designed for indoor use [41]. This highlights the critical need to complement such interventions with vector control strategies specifically tailored for outdoor and occupational settings [42].

When hazard, vulnerability, and elements at risk were integrated to generate the malaria risk map, hazard emerged as the most influential determinant of spatial risk patterns. This contrasts with the conclusions drawn by [39], who reported that elements at risk demonstrated higher influence on the weight in defining malaria risk zones. The current study established that in Nsanje, climatic and socio-spatial factors, particularly temperature, rainfall, and access to healthcare services, are more decisive than land cover characteristics. The sensitivity analysis corroborated these findings by highlighting hazard and vulnerability as the most sensitive components of the risk model. In contrast, elements at risk remained relatively stable under altered weighting conditions. These patterns suggest that malaria risk in Nsanje is primarily governed by environmental suitability and human accessibility to health systems rather than solely by the spatial distribution of susceptible land cover features. The proximity-to-health-facility analysis further indicates that health facilities are unevenly distributed across Nsanje District, leaving extensive rural areas in the high-distance classes. This spatial pattern suggests that a notable proportion of the population faces geographic barriers to timely diagnosis and treatment, which may contribute to sustained community transmission and higher case burdens in environmentally suitable zones.

The spatial distribution of risk revealed notable geographic variation across the district, with northern areas exhibiting higher malaria risk than southern regions. This pattern closely corresponded with the distribution of cumulative malaria incidence reported from 2014 to 2024, where facilities located within high-risk zones recorded the highest case burdens. This spatial agreement provides evidence that the model captures meaningful environmental and socio-spatial drivers of malaria transmission, consistent with previous work demonstrating strong associations between modelled environmental suitability and clinical malaria burden when geospatial approaches are applied at appropriate spatial scales [15, 20]. However, it is also important to interpret facility-reported incidence in light of healthcare access and reporting dynamics. In geographically remote areas, malaria burden may be under-detected or underreported due to reduced facility utilization, delayed care-seeking, and logistical challenges affecting surveillance completeness, a pattern that has been documented in other sub-Saharan African settings [43]. Therefore, the observation that the highest incidence clusters occur in mapped high-risk zones likely reflects a combination of genuinely elevated transmission potential and higher detection in areas where facilities are accessible enough to capture cases. This reinforces the operational value of the risk map for guiding targeted interventions, particularly in zones where both environmental suitability and service-access barriers intersect.

## Conclusions and recommendations

This study employs a heuristic GIS-based mapping approach rather than inferential spatial statistics. As such, it does not quantify spatial autocorrelation, model uncertainty, or establish statistical associations between risk factors and malaria outcomes. The weights assigned through the AHP reflect expert judgment synthesized from the literature rather than empirically derived relationships specific to Nsanje District. Based on expert weighting through AHP, climate factors (particularly temperature and rainfall) emerged as the most heavily weighted, while elevation, slope, and proximity to rivers were the less weighted components of the malaria hazard model, while elevation, slope, and proximity to rivers played lesser roles. Vulnerability was highest in areas furthest from health facilities, indicating that access to health care was a critical factor; population density and road proximity had a minimal influence on vulnerability. Farmland was moderately at risk, with the most susceptible areas being limited water bodies. The integration of hazard, vulnerability, and land-use data revealed that hazard was the primary driver of overall malaria risk, followed by vulnerability, which aligns with malaria case trends from 2014 to 2024. It is worth noting that the risk classifications in this study are as relative spatial prioritizations rather than absolute probability estimates. The absence of formal spatial statistical methods (e.g., Moran’s I for spatial autocorrelation, spatial regression models, or Bayesian geostatistical approaches) means that we cannot quantify the uncertainty around risk estimates or formally test hypotheses about spatial patterns. Future work would benefit from integrating these inferential approaches and climate projections, particularly if high-resolution malaria surveillance data with precise geocoding become available. The spatial patterns identified through this GIS-based mapping exercise provide an accessible tool for malaria control programme planning in Nsanje District. While our approach does not employ formal spatial statistical inference, the weighted overlay framework offers transparency and interpretability that may be particularly valuable for engaging stakeholders and guiding resource allocation decisions.

## Data Availability

The data used will be provided upon request to the authors.

## [Figure 1] Long description

Starting from the northwest, the map displays Nsanje district with elevation ranging from high (orange) in the west to low (purple) in the east and southeast. Health facility icons are distributed throughout, with clusters near main settlements. The main road, marked in red, runs north to south, intersecting several rivers shown in blue. District boundaries are outlined in black, while T A boundaries are in green. Major areas labeled include T A Mlolo in the north, S C Mbenje in the center, Mwabvi Game Reserve in the southwest, T A Tengani in the southeast, and multiple smaller administrative units in the south. The inset at the upper right shows the location of Nsanje within Malawi, adjacent to Lake Malawi. The scale bar at the bottom right indicates distances up to 20 kilometers. The legend at the lower left explains symbols for health facilities, roads, rivers, boundaries, and elevation gradient.

Navigate back to [Figure 1].

## [Table 1] Long description

Starting from the top row, the table has four columns: Data, Resolution, Purpose, and Source. The first row lists Temperature (Jan–Dec 2024) with resolution 0.5 degrees by 0.625 degrees, used for temperature mapping, sourced from NASA POWER. The second row is Rainfall with resolution 0.05 degrees, for rainfall mapping, sourced from CHIRPS. The third row is S R T M D E M with 30 meters resolution, used for elevation, slope, and river proximity mapping, sourced from U S G S. The fourth row is Roads, resolution not specified, for proximity to roads, sourced from OpenStreetMap. The fifth row is Health facilities, resolution not specified, for proximity to health facilities, sourced from Mo H. The sixth row is Population density, resolution not specified, for population density mapping, sourced from N S O. The seventh row is Sentinel–2 imagery with 10 meters resolution, for L U L C mapping, sourced from Copernicus (E S A). The eighth row is Malaria incidence, resolution not specified, for malaria risk comparison, sourced from D H I S 2. The ninth row is Administration boundaries, resolution not specified, for study area, country, and T A boundaries, sourced from D I V A G I S. Each source is either a named organization or a web link, with italic formatting for some sources.

Navigate back to [Table 1].

## [Figure 2] Long description

From the left, four data sources are listed vertically: S R T M D E M with rivers proximity, elevation, slope; Climate Data with rainfall, temperature; Socio-economic with population density, roads proximity, health facility proximity; Sentinel 2 with supervised classification. S R T M D E M and Climate Data both connect to Weight Assignment with A H P, then to Ranking, then to Weighted Overlay, producing the Hazard Map. Socio-economic data connects to Weight Assignment with A H P, then splits: one path leads directly to Vulnerability Map, the other to Ranking, then Weighted Overlay, which also produces the Vulnerability Map. Sentinel 2 data is processed by supervised classification, then Ranking, producing the L U L C Map. The L U L C Map and Vulnerability Map both connect to Weighted Assignment with A H P, then Weighted Overlay, resulting in the Risk Map.

Navigate back to [Figure 2].

## [Figure 3] Long description

Top-left panel labeled 1 shows temperature hazard, with very high levels in the north and very low in the south. Top-center panel labeled 2 shows rainfall hazard, with very high in the north and very low in the south. Top-right panel labeled 3 shows proximity to river, with very high hazard concentrated along central and southern river corridors, decreasing outward. Bottom-left panel labeled 4 shows elevation, with very high hazard in the north and very low in the south. Bottom-center panel labeled 5 shows slope, with very high hazard in the north and very low in the south. All panels use the same legend: yellow for very high, light green for high, green for moderate, blue-green for low, and blue for very low hazard. District boundaries are outlined in gray. A compass rose and scale bar are present in the bottom-right.

Navigate back to [Figure 3].

## [Table 2] Long description

The table contains five factors: Rainfall in millimeters, Temperature in degrees Celsius, Slope in degrees, Elevation in meters, and Proximity to Rivers in meters. For each factor, five classes are defined with corresponding ranges, ranks from 1 to 5, and hazard levels from very low to very high. Rainfall: 1,490 to 1,871 is very low hazard (rank 1), 1,117 to 1,490 is low (2), 743 to 1,117 is moderate (3), 376 to 743 is high (4), 3 to 376 is very high (5). Temperature: 19 to 21 degrees Celsius is very low (1), 21 to 22 is low (2), 22 to 23 is moderate (3), 23 to 24 is high (4), 24 to 25 is very high (5). Slope: 21 to 60 degrees is very low (1), 12 to 21 is low (2), 6 to 12 is moderate (3), 1 to 6 is high (4), 0 to 1 is very high (5). Elevation: 507 to 961 meters is very low (1), 331 to 507 is low (2), 203 to 331 is moderate (3), 104 to 203 is high (4), 28 to 104 is very high (5). Proximity to Rivers: 6,008 to 11,265 meters is very low (1), 3,799 to 6,008 is low (2), 2,121 to 3,799 is moderate (3), 707 to 2,121 is high (4), 0 to 707 is very high (5). Hazard increases as rainfall, temperature, slope, elevation, or proximity to rivers decrease within the specified ranges.

Navigate back to [Table 2].

## [Table 3] Long description

From top to bottom, the table lists three factors: Population Density in persons per square kilometer, Proximity to Health Facility in meters, and Proximity to Roads in meters. Each factor is divided into five classes, each with an associated rank from 1 to 5 and a vulnerability level. For Population Density: 25 to 40 is rank 1, very low vulnerability; 40 to 64 is rank 2, low; 64 to 169 is rank 3, moderate; 169 to 501 is rank 4, high; 501 to 1741 is rank 5, very high. For Proximity to Health Facility: 0 to 1,287 is rank 1, very low; 1,287 to 3,552 is rank 2, low; 3,552 to 5,612 is rank 3, moderate; 5,612 to 8,031 is rank 4, high; 8,031 to 13,128 is rank 5, very high. For Proximity to Roads: 0 to 1,694 is rank 1, very low; 1,694 to 5,271 is rank 2, low; 5,271 to 9,789 is rank 3, moderate; 9,789 to 15,248 is rank 4, high; 15,248 to 24,002 is rank 5, very high. Each factor’s classes increase in value and vulnerability from top to bottom.

Navigate back to [Table 3].

## [Figure 4] Long description

From left to right, the first panel maps vulnerability by health facility proximity, with concentric rings of color from yellow (very low) to red (very high) centered around hospitals, showing highest vulnerability in areas farthest from hospitals. The second panel maps vulnerability by population density, with the north and central regions shaded yellow and orange (very low to moderate), and the southern region in red (very high), indicating highest vulnerability in the south. The third panel maps vulnerability by road proximity, with bands of color parallel to main roads: yellow (very low) near roads, progressing to red (very high) farther away, with highest vulnerability along the district’s eastern and western edges. All panels include district boundaries, a north arrow, and a scale bar. Legends specify color codes for vulnerability levels: very low, low, moderate, high, and very high.

Navigate back to [Figure 4].

## [Table 4] Long description

The header row contains three columns: Element at Risk, Rank, and Susceptibility. The first row lists Vegetation, rank 1, susceptibility very low. The second row lists Bare or Grassland, rank 2, susceptibility low. The third row lists Farmland, rank 3, susceptibility moderate. The fourth row lists Settlement, rank 4, susceptibility high. The fifth row lists Water, rank 5, susceptibility very high. The susceptibility increases with rank from vegetation to water.

Navigate back to [Table 4].

## [Table 5] Long description

The table is divided into three sections. The first section, hazard criteria, lists 20 model runs in rows. Columns from left to right are model run, temperature percent, rainfall percent, slope percent, elevation percent, and river proximity percent. For example, model run 1 assigns 10 to temperature, 22.5 to rainfall, slope, elevation, and river proximity. Model run 4 assigns 70 to temperature and 7.5 to the other four criteria. The second section, vulnerability criteria, lists 12 model runs. Columns are model run, population density percent, hospital proximity percent, and road proximity percent. For example, model run 1 assigns 10 to population density, 45 to hospital proximity, and 45 to road proximity. Model run 4 assigns 70 to population density and 15 to both hospital and road proximity. The third section, risk factors, lists 12 model runs. Columns are model run, hazard percent, vulnerability percent, and elements at risk percent. For example, model run 1 assigns 10 to hazard, 45 to vulnerability, and 45 to elements at risk. Model run 4 assigns 70 to hazard and 15 to both vulnerability and elements at risk. All percentages are distributed to sum to 100 within each model run row.

Navigate back to [Table 5].

## [Figure 5] Long description

The map displays Nsanje District outlined in black, oriented with north at the top. Five malaria hazard levels are shown using distinct colors: very high (dark brown) dominates the northernmost area, high (brown) is just south of this, moderate (yellow) covers the central and southeastern regions, low (purple) appears in the southern third, and very low (green) is concentrated at the southern tip. The legend at the lower left explains the color scheme. A scale bar in the upper right indicates distances up to 20 kilometers. Grid coordinates are labeled along the map edges.

Navigate back to [Figure 5].

## [Table 6] Long description

From top to bottom, the table columns are Factor and Weight in percent. Rainfall is 34.9, temperature is 34.9, slope is 5.1, elevation is 10.7, and proximity to rivers is 14.3. Rainfall and temperature have the highest weights, slope the lowest.

Navigate back to [Table 6].

## [Figure 6] Long description

Starting at the northern boundary and moving south, the district is divided into overlapping circular zones colored blue for very low, light blue for low, yellow for moderate, orange for high, and red for very high vulnerability. Very low and low vulnerability zones, shown as blue and light blue circles, are scattered throughout the district, often overlapping. Moderate vulnerability, shown in yellow, forms the background and is the most widespread, filling most of the district except for the edges. High vulnerability, in orange, appears as irregular patches mainly in the central and southern regions. Very high vulnerability, in red, is concentrated along the eastern boundary, especially in the southeast and northeast corners. The district boundary is outlined in black. A legend at the lower left explains the color codes. A north arrow and scale bar are present in the upper left and right center, respectively. Grid coordinates are labeled along the map edges.

Navigate back to [Figure 6].

## [Table 7] Long description

From the top row downward, the table presents three factors with corresponding weights. Population density is assigned a weight of 25.8 percent. Health facility proximity has the highest weight at 63.7 percent. Road proximity is weighted at 10.5 percent. The columns are labeled Factor and Weight percent.

Navigate back to [Table 7].

## [Figure 7] Long description

The map displays Nsanje District outlined in black, oriented with north at the top. The legend at the lower left assigns green to vegetation (very low risk), yellow to farmland (moderate risk), orange to bare or grassland (low risk), red to settlement (high risk), and blue to water (very high risk). Vegetation is concentrated in the north and along the southern and eastern boundaries. Farmland, shown in yellow, dominates the central and southern interior. Bare or grassland, in orange, is scattered throughout but more prominent in the central region. Settlements, marked in red, are clustered in small patches mainly in the central and southern zones. Water bodies, in blue, are limited and primarily found in the north-central area. The map includes a scale bar (0 to 20 kilometers) and grid coordinates along the borders. The compass rose in the northwest corner indicates cardinal directions.

Navigate back to [Figure 7].

## [Table 8] Long description

From the top row downward, the table columns are Element at risk, Area in square kilometers, and Percentage. Vegetation covers 341.5 square kilometers at 17.6 percent. Bare or grassland covers 675.9 square kilometers at 34.9 percent. Farmland covers 847.6 square kilometers at 43.7 percent. Settlement covers 57.9 square kilometers at 3.0 percent. Water covers 16.2 square kilometers at 0.8 percent. The spatial hierarchy follows the order of L U L C classes as listed.

Navigate back to [Table 8].

## [Figure 8] Long description

Panel a on the left displays a district map with malaria risk categorized by color: very low in pale yellow, low in light orange, moderate in orange, high in red, and very high in dark red. The highest risk is concentrated in the northern and central districts, with lower risk in the southern region. District boundaries are outlined in gray. A scale bar and compass rose are present. Panel b on the right overlays the same risk map with blue circles of varying sizes, each representing cumulative malaria cases per health facility from 2014 to 2024. Larger circles, indicating higher case counts, are clustered in the north and central districts, aligning with areas of high and very high risk. The legend for circle size ranges from 1,000 to 100,000 cases. Both panels use identical color scales and district boundaries for direct comparison.

Navigate back to [Figure 8].

## [Figure 9] Long description

Panel a, top-left, shows temperature sensitivity. For 70 percent weighting, very low is 40.96, moderate is 28.63, high is 18.23, low is 10.54, very high is 1.64. For 50 percent, very low is 41.76, moderate is 23.76, high is 10.58, low is 10.54, very high is 0.53. For 30 percent, very low is 44.11, moderate is 20.94, high is 7.57, low is 20.94, very high is 0.22. For 10 percent, very low is 46.49, moderate is 23.64, high is 8.71, low is 7.81, very high is 0.22. Panel b, top-center, shows rainfall sensitivity. For 70 percent, very low is 68.28, moderate is 24.84, high is 16.98, low is 2.94, very high is 2.94. For 50 percent, very low is 35.17, moderate is 17.47, high is 28.03, low is 18.53, very high is 15.46. For 30 percent, very low is 38.38, moderate is 20.94, high is 8.97, low is 31.49, very high is 0.22. For 10 percent, very low is 48.27, moderate is 22.98, high is 21.96, low is 6.58, very high is 0.22. Panel c, bottom-left, shows elevation sensitivity. For 70 percent, very low is 34.26, moderate is 35.74, high is 15.85, low is 11.42, very high is 2.73. For 50 percent, very low is 43.35, moderate is 26.22, high is 10.08, low is 19.46, very high is 0.88. For 30 percent, very low is 49.68, moderate is 20.94, high is 8.10, low is 20.94, very high is 0.22. For 10 percent, very low is 40.99, moderate is 30.49, high is 20.99, low is 7.32, very high is 0.22. Panel d, bottom-center, shows slope sensitivity. For 70 percent, very low is 44.67, moderate is 34.28, high is 11.89, low is 7.55, very high is 1.61. For 50 percent, very low is 47.73, moderate is 28.54, high is 15.39, low is 7.71, very high is 0.64. For 30 percent, very low is 50.01, moderate is 21.20, high is 7.64, low is 20.93, very high is 0.22. For 10 percent, very low is 40.31, moderate is 30.30, high is 8.18, low is 20.93, very high is 0.25. Panel e, bottom, shows river proximity sensitivity. For 70 percent, very low is 37.70, moderate is 26.45, high is 26.48, low is 9.31, very high is 0.05. For 50 percent, very low is 44.50, moderate is 20.45, high is 29.73, low is 5.28, very high is 0.03. For 30 percent, very low is 47.03, moderate is 27.88, high is 18.24, low is 6.69, very high is 0.16. For 10 percent, very low is 43.46, moderate is 24.50, high is 22.45, low is 9.14, very high is 0.45. Each panel uses the same color scheme for sensitivity classes: red for very high, orange for high, yellow for moderate, green for low, and brown for very low. The trend across all panels is that very low sensitivity generally has the highest areal coverage, especially at higher weighting levels.

Navigate back to [Figure 9].

## [Figure 10] Long description

Panel a at top left shows population density. For 70 percent weighting, very high is 0, high 10.17, moderate 12.97, low 37.22, very low 39.64. For 50 percent, very high 0, high 5.31, moderate 6.67, low 46.10, very low 41.92. For 30 percent, very high 0, high 3.89, moderate 9.07, low 42.29, very low 44.74. For 10 percent, very high 0, high 14.69, moderate 17.44, low 33.03, very low 34.85. Panel b at top right shows proximity to health facility. For 70 percent weighting, very high 0.44, high 16.03, moderate 13.81, low 34.39, very low 35.32. For 50 percent, very high 0, high 13.14, moderate 5.47, low 40.47, very low 40.92. For 30 percent, very high 0, high 3.89, moderate 9.07, low 42.29, very low 44.74. For 10 percent, very high 0, high 3.34, moderate 10.43, low 38.61, very low 47.63. Panel c at bottom center shows proximity to roads. For 70 percent weighting, very high 0.01, high 14.16, moderate 14.16, low 28.40, very low 33.88. For 50 percent, very high 0.01, high 8.93, moderate 9.04, low 40.48, very low 41.53. For 30 percent, very high 0, high 3.89, moderate 9.07, low 42.29, very low 44.74. For 10 percent, very high 0, high 5.00, moderate 11.25, low 40.81, very low 42.94. Color legend at top left: red for very high, brown for high, yellow for moderate, light green for low, dark green for very low.

Navigate back to [Figure 10].

## [Figure 11] Long description

Panel a at left shows hazard with five bars for 10, 30, 50, and 70 percent weighting. At 10 percent, moderate is 38.73, low is 58.63, very low is 0.35, high is 2.23, very high is 0.06. At 30 percent, moderate is 21.21, low is 74.49, very low is 4.11, high is 0.15, very high is 0.03. At 50 percent, moderate is 25.49, low is 60.40, very low is 13.79, high is 0.17, very high is 0.15. At 70 percent, moderate is 14.30, low is 41.11, very low is 42.93, high is 1.00, very high is 0.65. Panel b at top right shows vulnerability. At 10 percent, moderate is 19.87, low is 68.08, very low is 11.35, high is 0.43, very high is 0.28. At 30 percent, moderate is 21.21, low is 74.49, very low is 4.11, high is 0.03, very high is 0.15. At 50 percent, moderate is 19.57, low is 71.00, very low is 9.39, high is 0.05, very high is 0.00. At 70 percent, moderate is 10.77, low is 42.16, very low is 46.67, high is 0.37, very high is 0.02. Panel c at bottom shows elements at risk. At 10 percent, moderate is 18.46, low is 70.75, very low is 10.61, high is 0.03, very high is 0.15. At 30 percent, moderate is 21.21, low is 74.49, very low is 4.11, high is 0.03, very high is 0.15. At 50 percent, moderate is 23.84, low is 71.21, very low is 4.61, high is 0.18, very high is 0.15. At 70 percent, moderate is 4.40, low is 46.17, very low is 46.01, high is 0.50, very high is 0.50. Across all panels, moderate and low risk categories dominate area coverage, with very high and high risks consistently below 2.5 percent.

Navigate back to [Figure 11].
